# Host Response to Biomaterials for Cartilage Tissue Engineering: Key to Remodeling

**DOI:** 10.3389/fbioe.2021.664592

**Published:** 2021-05-04

**Authors:** Fu Wei, Shuyun Liu, Mingxue Chen, Guangzhao Tian, Kangkang Zha, Zhen Yang, Shuangpeng Jiang, Muzhe Li, Xiang Sui, Zhiwei Chen, Quanyi Guo

**Affiliations:** ^1^Beijing Key Lab of Regenerative Medicine in Orthopedics, Key Laboratory of Musculoskeletal Trauma and War Injuries, PLA, Institute of Orthopedics, Chinese PLA General Hospital, Beijing, China; ^2^Department of Orthopedics, The First Affiliated Hospital of University of South China, Hengyang, China; ^3^Department of Orthopedic Surgery, Beijing Jishuitan Hospital, Fourth Clinical College of Peking University, Beijing, China; ^4^School of Medicine, Nankai University, Tianjin, China; ^5^The First Hospital of China Medical University, Shenyang, China

**Keywords:** biomaterials, immunomodulation, macrophage, tissue engineering, cartilage

## Abstract

Biomaterials play a core role in cartilage repair and regeneration. The success or failure of an implanted biomaterial is largely dependent on host response following implantation. Host response has been considered to be influenced by numerous factors, such as immune components of materials, cytokines and inflammatory agents induced by implants. Both synthetic and native materials involve immune components, which are also termed as immunogenicity. Generally, the innate and adaptive immune system will be activated and various cytokines and inflammatory agents will be consequently released after biomaterials implantation, and further triggers host response to biomaterials. This will guide the constructive remolding process of damaged tissue. Therefore, biomaterial immunogenicity should be given more attention. Further understanding the specific biological mechanisms of host response to biomaterials and the effects of the host-biomaterial interaction may be beneficial to promote cartilage repair and regeneration. In this review, we summarized the characteristics of the host response to implants and the immunomodulatory properties of varied biomaterial. We hope this review will provide scientists with inspiration in cartilage regeneration by controlling immune components of biomaterials and modulating the immune system.

## Introduction

Articular cartilage lacks blood vessels, nerves and lymphatic vessels, has a smooth surface and is translucent. It is a form of hyaline cartilage, which is a highly organized connective tissue (Sophia-Fox et al., 2009). Articular cartilage bears mechanical loads and provides cushioning and lubrication, and its protective function is weakened after injury. Due to the lack of self-healing capacity of cartilage, joint injury and progressive degeneration, osteoarthritis (OA) eventually develops, and it becomes difficult to avoid joint arthroplasty (Widuchowski et al., 2007). Therefore, the early repair of damaged articular cartilage is necessary.

Non-operative treatment often involves oral glucosamine sulfate to nourish cartilage and intra-articular injections of Hyaluronic acid (HA) to lubricate the joint. Traditional surgical treatment includes abrasion, subchondral drilling, microfracture, osteochondral autograft transfer system (OATS) (also known as mosaicplasty), and autologous chondrocyte implantation (ACI) (Pestka et al., 2012; Albright and Daoud, 2017; Solheim et al., 2018). However, these methods have achieved limited success. HA offers little protection against early cartilage damage (Nathani et al., 2019). Microfracture is used to treat small articular cartilage lesions that form fibrocartilage and are therefore mechanically weak and prone to degeneration (Benthien and Behrens, 2013). Mosaicplasty is usually suitable for the damage involving subchondral bone, but donor area complications and dead spaces between cylindrical grafts may increase the chance of repair failure (Kock et al., 2008). ACI is a cell-engineering base surgical procedure, but the quality of restoration is severely compromised by the loss of therapeutic chondrocytes (McCarthy et al., 2016). There is an urgent clinical need for new strategies to induce cartilage regeneration. Tissue engineering has emerged in recent years as a promising approach to repair injured cartilage (Makris et al., 2015), aiming to exploit the inherent regenerative potential of human tissues and organs in the degraded state and restore or reconstruct the normal function of the body.

As an indispensable element of the tissue engineering triad, biomaterials not only provide mechanical support for cells, but also act as carriers for bioactive molecules (e.g., growth factors). The biomaterials used to fabricate scaffolds can be roughly divided into two main categories: synthetic polymers, including polycaprolactone (PCL), poly(lactic acid) (PLA), poly(glycolic acid) (PGA), poly(lactic-co-glycolic acid) (PLGA), and poly(L-lactic acid) (PLLA), among others. Alternatively, naturally derived biomaterials are for instance decellularized extracellular matrix (dECM) from diverse tissues and natural molecules such as collagen, gelatin, silk fibroin, HA, alginate, and chitosan, et al. These materials can be used to fabricate different geometric shapes by 3D printing, electrospinning, injection molding techniques and among others (Chen et al., 2018; Lukanina et al., 2018; Xia et al., 2018; Lu et al., 2019). The scaffolds fabricated using these two kinds of biomaterials have advantages and disadvantages (Londono and Badylak, 2015). Compared with synthetic polymers, naturally derived biomaterials have a composition closer to that of cartilage, good biocompatibility and low cytotoxicity, and they are beneficial for cell adhesion, proliferation and differentiation (Huang et al., 2019; Xiao et al., 2019). However, these materials are limited in terms of processability and manipulatable degradation time (Pati et al., 2014). In contrast, synthetic polymers have good mechanical strength, controllable degradation and high plasticity (Li et al., 2019b; Stefani et al., 2020). But they also have shortcomings, such as poor biocompatibility, acidic degradation products, low biological activity, hydrophobicity and limited interfacial integration with tissue (Stocco et al., 2014; Park et al., 2018; Lin et al., 2019).

The great potential of tissue engineering for cartilage repair has given rise to new hope. As mentioned above, a wide variety of biomaterials have been researched and developed to achieve better repair and regeneration outcomes. But up to now, when implanted into vivo, most of the biomaterials have not worked as expected, some even lead to acute or chronic inflammation (Cipriano et al., 2017; Vollkommer et al., 2019). An important link seems to have been ignored by us. Numerous researches on cartilage tissue engineering are focused on the effect of biomaterial’s physical properties and physicochemical properties on repair outcome. However, limited studies have focused on how biomaterials affect host response, such as inflammation and immune modulation. Studies have suggested that implanted biomaterial induced adverse immune response is associated with a prolonged reaction time, delayed interface connection and integration, and reconstruction failure (Sirlin et al., 2001; Fraitzl et al., 2008). It has also been shown that the host immune response has a positive effect on the reparative effect of tissue engineering scaffolds to some extent (Sadtler et al., 2019). Direct use of acellular extracellular matrix (ECM) or native ECM components can elicit a favorable immune response prior to tissue remodeling outcome. This immunomodulation usually depends on the composition and structure of the scaffold. Tissue-derived ECM scaffolds induce a pro-regeneration immune environment through Th2 immune response, which drive macrophage polarization toward M2 phenotype via IL-4 (Cipriano et al., 2017). Besides, the regulatory role of pore size and porosity of scaffold in host response has also been confirmed (Orenstein et al., 2010; Junge et al., 2012). In scaffold strategy based on loading immunomodulatory function cells, mesenchymal stem cells (MSCs), as commonly used seed cells, can regulate local immune response by secreting anti-inflammatory and pro-inflammatory cytokines to promote repair (English, 2013; Nagubothu et al., 2020). Indeed, the interaction between implanted biomaterial and host immune system is crucial in determining the constructive and functional outcome. Host response is considered to be influenced by the immune components (also known as immunogenicity) of biomaterial, cytokines, or inflammatory agents induced by implants and injured tissue. As such the immunogenicity of implanted biomaterials should not be ignored. Additionally, cartilage is believed to be a tissue with “immune privilege,” located in a relatively closed environment (Smith et al., 2015). Nevertheless, cartilage tissue injury is accompanied by the disruption of balanced environment, release of inflammatory cytokines and chemokines, and immune/inflammatory cells migration will further promote the response of the host immune system to the implanted biomaterial.

The host response to biomaterials plays a central role in tissue repair and regeneration, have both positive and negative implication for the healing process. An effective biomaterial-based immune modulation strategy is becoming an attractive approach in the field of regenerative medicine (Hachim et al., 2019; Shahbaz et al., 2020; Wu et al., 2020). This review summarizes the characteristics of the interaction between host immune system and implanted biomaterial, and the immunomodulatory properties of common biomaterials in the hope of providing inspiration for next generation of tissue engineering regeneration strategy.

## Host Response Following Biomaterial Implantation

Biomaterials implanted into damaged tissues come into contact with cells, surrounding tissues and blood and thus cannot avoid activating the immune system by triggering a foreign body reaction. In fact, the relationship between biomaterial-mediated tissue healing and immune response is very complex, with many cell types, plasma proteins, and extracellular fluid components being involved. The reaction of the biomaterial with the surrounding environment is actually an important factor in determining its biocompatibility. The host response (including immune, inflammatory and foreign body reactions, etc.) can be local or systemic, which is mainly influenced by immune components of the implant, type of biomaterial or injured tissue, and biomaterial surface chemistry and degradation characteristics, and so on. This will ultimately affect the lifespan of the implant and the outcome of the repair.

### The Vroman Effect of Implanted Biomaterials

**FIGURE 1:**
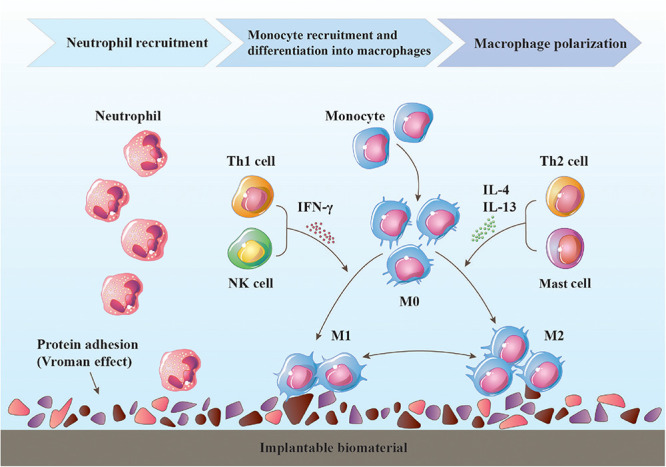
Immune response to an implantable biomaterial. Various proteins adhere to the surface of the biomaterial. Neutrophils are first recruited, followed by monocyte-derived macrophages, which become the dominant cell type around the biomaterial. Macrophage differentiation is temporary, and the polarization state may change depending on the tissue milieu. The timely transition of the macrophage phenotype from proinflammatory M1 to anti-inflammatory and immunomodulatory M2 is critical for constructive tissue remodeling, and other immune cells (T helper cells, NK cells, mast cells, etc.) are also involved in this process.

Biomaterial-mediated host reactions are primarily caused by host cell recognition of the biomaterial surface, which is based on the adsorption of adhesion proteins to the surface of the biomaterial. Adhesion proteins, such as fibrinogen, vitronectin, fibronectin, albumin, γ globulin, complement, and others, form a protein layer on the surface of the biomaterial to modulate host inflammatory cell interactions and adhesion (Pape et al., 2017; Tanaka et al., 2017; Horbett, 2018). The process of cell adsorption to and spreading on the biomaterial surface is mediated by adhesion proteins. The effect of a biomaterial on the cell or host reaction is actually achieved by influencing the adhesion behavior of proteins to the surface. The type, concentration and spatial conformation of the adhesion protein will directly activate the response of the host immune cell to the biomaterial (Wilson et al., 2005), i.e., resulting in an innate or adaptive immune response. In turn, the type, concentration and spatial conformation of these adsorbed proteins depend on the surface characteristics of the biomaterial, which determine the adsorption, survival and function of cells (especially monocytes and macrophages) in the protein layer (Anderson et al., 2008). Protein adhesion is a dynamic process, a high concentration of adhesion proteins first arrive and adsorb onto the surface of the implant, and these proteins will eventually be replaced by proteins with a high affinity for the surface of the biomaterial. The phenomenon of protein adsorption and desorption is known as the Vroman effect (Figure 1; Hirsh et al., 2013; Kim, 2017). After implantation, biomaterial is immediately covered by proteins in the blood and tissue fluid, which direct the subsequent cascade biological reactions. Studies have shown that the orientation and conformation of fibrinogen on the surface of biomaterials can modulate neutrophils adhesion (Milleret et al., 2015). There is a direct relationship between a stronger adhesion capacity on the surface of biomaterials and more desirable cell adhesion and growth (Shamloo and Sarmadi, 2016). It is well known that at similar surface roughness, hydrophilic surfaces preferentially adsorb proteins and promote cell adhesion and growth compared to hydrophobic surfaces (Chaudhary et al., 2012). By immobilizing proteins on the biomaterial surface, developing a biointerface that can enhance cell “homing” ability is one of the critical factors in biomaterial development (Chrzanowski et al., 2012). The biomaterial surface properties, protein adsorption and cellular responses are considered to be interrelated and ultimately determine the biocompatibility of the biomaterial (Allen et al., 2006). The relationship between these different but related phenomena remains to be elucidated.

### The Main Actors in Host-Biomaterial Interaction

There is a class of integrin receptors on the cell surface that can specifically recognize certain peptides of adhesion proteins, thereby affecting cell adhesion, migration, activity, and other functions (Yang et al., 2013). The type and level of cell adhesion proteins change in a time-dependent manner (Xu and Siedlecki, 2007). The early stage of host response following tissue injury is characterized by a predominance of neutrophils, which are the first inflammatory cell type to arrive at the site of injury. Neutrophils are capable of phagocytosis and degradation of foreign substances, dead cells, and providing signaling molecules to recruit macrophages to injury site. Mast cells modulate the inflammatory response by releasing particles, such as histamine, IL-4, and IL-13 that can recruit macrophages and facilitate their fusion into foreign body giant cells (DeFife et al., 1997; Ang et al., 1998; McNally and Anderson, 2002). Macrophages begin to infiltrate approximately 12 h post-injury, but their response lasts longer than that of neutrophils, and subsequently become the dominant cell type within the site of injury. The role of macrophages in tissue repair and regeneration is very complex, could perform beneficial or detrimental functions. That is, deciding toward to a constructive and functional outcome or scar tissue formation. The T lymphocyte cell population plays an important regulatory role in tissue repair by the secretion of cytokines and chemokines, many of which are considered to influence macrophage polarization. Moreover, dendritic cells, natural killer (NK) cells, B lymphocytes, plasmocytes, and among others also participate in the immune response to varying degrees (Figure 1). The occurrence and outcome of a series of events will vary according to the surface chemistry, stiffness, and degradation properties of biomaterials, as well as other factors that have not been elucidated (Brodbeck et al., 2001).

With the advances in tissue engineering and regeneration medicine, it has now become clear that the immune system plays a critical role in tissue repair process (Julier et al., 2017). Repair strategy of immune system regulated through biomaterials is aim at activating desired components of inflammatory, proliferation, and remolding phase to promote a constructive and functional remolding outcome. Allowing specific biological response is beneficial to both the integration and performance, the current challenge is the development of biomaterials or delivery systems capable of modulating the immune system as a way of stimulating tissue repair and regeneration (Badylak et al., 2008). Therefore, a deeper understanding of immunological profile of biomaterials and the interaction of immune system and biomaterials interaction is essential for the better repair outcomes.

## Immunological Characterization of Biomaterials

### Synthetic Polymers

In the body, articular cartilage is subjected to multiaxial loads, including those resulting from compression, tension, shear and fluid flow (Pattappa et al., 2019). Articular cartilage exhibits excellent biomechanics due to its unique hyaline-like cartilage composition and the ultrastructure of the ECM, especially the tight network of type II collagen (Col II) fibrils and abundant negatively charged proteoglycan chains. The controlled mechanical properties of synthetic polymers can meet the biomechanical requirements of the process of cartilage regeneration (Silva et al., 2020). Synthetic polymers have good plasticity, and their microstructure, morphology, and degradation rate can be predesigned and regulated according to the biology of specific tissue (Wang et al., 2020b). PCL (Venugopal et al., 2019), PLA (Baena et al., 2019), PGA (Lin et al., 2017), and PLGA (Kim et al., 2019) are the most representative and widely used synthetic polymers, and have been approved by the FDA for clinical human use. Scaffolds made from synthetic polymers temporarily provide mechanical support after implantation, as well as a microenvironment for cell growth, proliferation and differentiation, thereby regulating and inducing tissue differentiation and regeneration (Li S. et al., 2020; Salonius et al., 2020). The structure of the scaffold is very important for cartilage regeneration, and tissue engineering applications often require the use of porous three-dimensional (3D) scaffolds to facilitate cell migration and cell-cell interactions (Schipani et al., 2020). Secondly, during the process of regeneration, scaffold should also have sufficient strength to resist the physiological load, and the stress should be properly distributed to the surrounding tissue (Lohfeld et al., 2015). The good printing properties of synthetic polymers allow their combination with 3D printing techniques to effectively address this issue. The controllability, reproducibility and good mechanical properties of synthetic polymers show promising applications in cartilage tissue engineering. However, the lower bioactivity they exhibit is not favorable for incorporating with host tissues, as they may lead to increased local pH through acidic degradation products, immune response or toxicity, and inflammation associated with high molecular weight polymers.

#### Acidic Degradation Products of Synthetic Polymers

Synthetic polymers have one common feature: degradability. Degradation occurs progressively (by chemical hydrolysis or enzymatic degradation) over time after implantation. Acidic degradation products reduce the pH of the surrounding environment, leading to inflammatory cell infiltration and aseptic inflammation (Lendlein and Langer, 2002). Clinical reports have shown that up to approximately 8% of patients treated with PGA had non-infectious inflammation (Peppas and Langer, 1994). The longer the biodegradation cycle of synthetic polymers, the greater the probability of inducing an adverse host response, this event is related to the acidic degradation products and immunogenicity of the polymer. Acidic products (e.g., lactic acid, glycolic acid) will trigger an autocatalytic reaction when reducing the pH, which can accelerate the formation of the products (Göpferich, 1996; Burkersroda et al., 2002; Eglin and Alini, 2008). When the accumulation of acidic products exceeds the metabolic capacity of the body, the gradually decreasing acidity of the environment in turn accelerates the degradation of the biomaterial. These acidic products are one of the factors involved in the host-biomaterial reaction (Rezwan et al., 2006). In addition, the surface chemistry, structure, type, purity, and crystallinity of the biomaterials also influence the occurrence, extent and outcome of the host-biomaterial interaction (Milleret et al., 2015; Jiang et al., 2016; Stratton et al., 2016).

#### Surface Chemistry: Hydrophobicity/Hydrophilicity, Chemical Groups, Charge Characteristics

As previously mentioned, the adsorption of proteins on biomaterial surface is essential for the cell-biomaterial interaction (Ekdahl et al., 2012). A factor that directly determines the type, amount and conformation of adhesion proteins is the biomaterial surface chemistry, such as its hydrophobicity/hydrophilicity, chemical moieties and charge characteristics. By changing the biomaterial surface chemistry to regulate the adsorption of proteins, it has been shown that hydrophilic and anionic chemistries are more likely to induce macrophage apoptosis than hydrophobic or cationic surfaces, which may result in no inflammatory outcome (Brodbeck et al., 2001). Macrophages play an important and complex role in the regulation of the immune microenvironment of implant, moreover, the adhesion and activation of lymphocytes also depend on macrophages. Macrophage plasticity allows their adaptation to different stimuli (Rostam et al., 2016). M1 macrophages can drive the inflammatory response, causing the scaffold to be wrapped in fibrous tissue, separating it from the surrounding tissue (Fujihara et al., 2020). In contrast, M2 macrophages have anti-inflammatory properties and promote repair (Ma et al., 2014; Hotchkiss et al., 2018; Deng et al., 2020).

The commonly used synthetic polymers are hydrophobic, with a surface that has a high affinity for many kinds of proteins. When protein molecules adsorb onto the surface of a hydrophobic biomaterial, their conformation changes through hydrophobic interactions, exposing the hydrophobic domain and facilitating close binding to the surface (Lu and Park, 1991; Hu et al., 2001; Collier and Anderson, 2002; Heuberger et al., 2005; Evans-Nguyen et al., 2006). There is a direct correlation between the hydrophobicity of biomaterials and activation of the host immune system (Hu et al., 2001; Moyano et al., 2012). The conformational change of adhesion proteins on the biomaterial surface may be one of the reasons for the occurrence of adverse response. For instance, the foreign body reaction, inflammation, and the exposure of hidden structures and sequences of adhesion proteins allows them to act as receptors for the binding of inflammatory or immune cells to the surface (Thevenot et al., 2008). Innate immune cells are able to recognize invaders and induce an immune response through pattern recognition receptors (PRPs) (Brubaker et al., 2015), based on the combination of PRPs and ligands [pathogen-associated molecular patterns (PAMPs) (Janeway, 1989); damage-associated molecular patterns (DAMPs) (Matzinger, 1994)]. Hydrophobicity is considered to be a DAMP, in other words, the hydrophobic property of a biomaterial is its inherent immunogenicity, which may initiates constructive remolding or scar tissue formation (Seong and Matzinger, 2004). It has been found that hydrophobic biomaterial surfaces selectively interact with CD8+ T lymphocytes, while hydrophilic/neutral surfaces tend to interact with CD4+ T lymphocytes (Chang et al., 2009). The hydrophilicity and hydrophobicity of a biomaterial can regulate the behavior of immune cells (Brodbeck et al., 2002; Jones et al., 2007), and the effects on tissue repair need to be further explored. To minimize adverse events triggered by the immunogenic of hydrophobic biomaterial surfaces, the hydrophilic molecules polyethylene oxide (PEO) and polyethylene glycol (PEG) are often used as biomaterial coatings to reduce surface protein adhesion and increase hydrophilicity (Tiller et al., 2001; Drury and Mooney, 2003). These coatings can also prevent implantation-related infection and biofilm formation (Busscher et al., 2012). It has also been reported that the hydrophilicity of biomaterial can be improved by adjusting the content of graphene oxide (GO), which improved in correspondence to an increase of GO (Aidun et al., 2019; Jabari et al., 2019).

Chemical groups are another important surface characteristic of biomaterials, with amino (-NH_2_), carboxyl (-COOH), hydroxyl (-OH), and methyl (-NH_3_) groups being commonly explored. These chemical moieties have a significant effect on the host response to biomaterials. Surfaces with hydrophilic -NH_2_ or -OH groups (positively and neutrally charged, respectively) strongly stimulated inflammatory cell recruitment and the fibrotic response *in vivo* (Kamath et al., 2008). While surfaces with hydrophobic, neutral -NH_3_ groups induced a more severe inflammatory response, and hydrophilic surfaces with -COOH groups (negatively charged) resulted in reduced cell infiltration and a milder inflammatory reaction (Barbosa et al., 2003, 2006; Barbosaa et al., 2004; Nair et al., 2008). These different results suggest that the -NH_3_ surface can eliminate the adsorption of leukocytes and reduce the immune reaction (Sperlinga et al., 2005). It has also been shown that -NH_2_ groups shift macrophage polarization toward an anti-inflammatory M2 phenotype and decrease the number of proinflammatory M1 macrophages, while -COOH groups yield the opposite result (Bartneck et al., 2010). This suggests that these findings cannot be explained by a single experimental model and may vary *in vivo* and *in vitro*. Based on the existing knowledge, studies have begun to use chemical groups to modify the surface of engineered scaffolds to facilitate repair and regeneration.

A scaffold surface was modified with either -NH_2_ or -COOH, and it was found that both modified scaffolds could promote the adhesion and proliferation of adipose-derived stem cells (ADSCs). The difference was that scaffolds functionalized with -NH_2_ moieties promoted the osteogenic differentiation of ADSCs, while those functionalized with -COOH moieties promoted chondrogenic differentiation (Griffin et al., 2017). In terms of the ability to induce cell differentiation, -OH groups showed the strongest ability to induce osteogenic differentiation, followed by -NH_2_, -COOH, and -NH_3_ groups. Among them, -NH_3_ groups appeared to induce moderate myogenic differentiation (Keselowsky et al., 2004; Lan et al., 2005). Further studies revealed that -NH_2_-modified surfaces activate the extracellular signal-related kinase (ERK1/2) signaling pathway by promoting the expression of bone marrow stromal cell (BMSC) integrins to induce osteogenic differentiation (Bai et al., 2013). Further expanding the understanding of biomaterial surface characteristics and how biomaterials interact with host immune system is needed to more effectively repair damaged tissues (Damanik et al., 2014). In fact, biomaterial-mediated tissue repair and regeneration is influenced by a variety of factors, such as immune components of implant, immune milieu. The properties of biomaterials need to be more comprehensively understood for improved application.

### Natural Biomaterials

The other type of biomaterials is natural biomaterials, including decellularized tissues, natural polymers, and cell-derived matrix. Compared to synthetic polymers, they are derived from biological tissues and are more suitable for cell adhesion, proliferation, differentiation and so on. Secondly, there are no acidic products. Natural polymers include polysaccharides (such as HA, alginate, and chitosan) and proteins (gelatin, silk fibroin, and fibrin), are widely used in the production of scaffolds for cartilage regeneration. Due to their origins, These materials are characterized by high biocompatibility, bioactivity, and the degradation products are non-toxic; but, their low mechanical stability, rapid degradation, and poor stability greatly limits their applications (Wasyłeczko et al., 2020). For example, alginate based hydrogel scaffolds can support the growth and proliferation of enveloped chondrocytes and maintain their chondrocyte morphology, but they have poor stability and loss of mechanical strength in a short period of time (Bao et al., 2020). Secondly, alginate has low cell adhesion and cell interaction ability. Compared with other natural synthetic polymers, the main advantage of silk fibroin is its good strength and toughness, which is more suitable for the preparation of load bearing tissue engineering such as cartilage regeneration (Kundu et al., 2013). Gelatin was modified by methacrylate anhydride, and the prepared product methacrylamide enhanced the mechanical properties and degradation rate of gelatin, which makes it play an important role in the application of cartilage tissue engineering (Han et al., 2017). Unfortunately, these natural polymers are difficult to achieve hyaline cartilage regeneration, but rather non-valuable fibrocartilage. Natural biomaterials are favored by a wide range of researches, especially naturally derived ECM biomaterials. Decellularized ECM is considered to be the best choice because it can provide a microenvironment similar to natural ECM and further modulate the cellular behavior and function.

#### Rationale for Using Extracellular Matrix (ECM) as Biomaterial

The optimal scaffold biomaterial should be able to provide an environment like that in which tissue-resident cells survive, i.e., the ECM. It is a precise and orderly structural network composed of large molecules such as proteins and polysaccharides. The ECM provides physical structure, and the basic biochemical and biomechanical signals for regulating tissue morphology, differentiation and homeostasis (Wang et al., 2018; Nie et al., 2020). Tissue engineering technology is to mimic the structure and composition of the damaged tissue, providing an optimal environment for cell survival, cell-cell and cell-tissue interactions and signal transduction. The fabrication of functionalized scaffolds with biological activity made of natural biomaterials is undoubtedly a promising approach (Feng et al., 2020; Vainieri et al., 2020). Natural biomaterials such as dECM and natural polymers (collagen, silk fibroin, alginate, and chitosan) show repair potential in animal models of cartilage lesion (Dai et al., 2019; Pérez-Silos et al., 2019; Singh et al., 2019). Tissue (or organ) from various species after decellularization can be utilized for the repair of a damaged tissue (Sun et al., 2018; Lindberg et al., 2019). Such as there are studies have successfully used acellular human umbilical cord Wharton’s jelly as a biomaterial to repair articular cartilage (Zhao et al., 2018).

Natural biomaterials are being explored and applied increasingly extensively. The mechanism of the natural biomaterial-driven tissue remolding remains to be elucidated, and adverse immune responses after implantation is a great challenge hindering application (Wu et al., 2019). Because of natural composition and structure, natural biomaterials elicit different host responses than others. There is a growing body of evidence suggesting that the host immune response to a biomaterial not only affects its function but must also be the primary factor determining the success of the repair. The issue of the host immune response induced by natural biomaterials, especially dECM, is reviewed below.

#### Remove Immune Components via Decellularization

In recent decades, the decellularization of tissues (or organs) has been developed and applied as an emerging technology in tissue engineering and regenerative medicine. The main purpose of the decellularization process is to remove immune components (such as cells and nuclear materials), while preserving the natural structure and biochemical components. Acellular ECM has good biocompatibility and biological activity. Similar to the natural matrix environment, acellular ECM can regulate the biological function of resident cells and multifunctional stem cells and promote the recovery of the structure and function of the damaged tissue (Agrawal et al., 2010; Li et al., 2018). In addition to its application in the repair of articular cartilage, acellular ECM has been applied in various tissues (or organs) such as bone (Huber et al., 2017), tendon (Zhang S. et al., 2018), nerve (Chen et al., 2019), blood vessels (Gong et al., 2016), cornea (Chakraborty et al., 2019), and skin (Milan et al., 2019), and some success in achieving repair.

#### Evaluate the Decellularize Degree of ECM

The host immune response to natural biomaterial can be activated by cell surface markers, residual DNA, alpha-Gal epitopes, and major histocompatibility complexes (MHCs), among others (Sutherland et al., 2015). Few researchers have studied the immunogenicity of allogeneic or xenogeneic cartilage-derived biomaterials. Cartilage tissue is considered to be “immune privilege” and does not elicit an host immune response. Studies have shown that unsatisfying outcomes are still caused by cartilage-derived biomaterials *in vivo*. The decellularization of tissues (or organs) is typically achieved using one or a combination of the following approaches: chemical (Ventura et al., 2019), physical (Schneider et al., 2016), and enzymatic (Luo et al., 2015; Li et al., 2019c). Decellularization technology broadens the source of biomaterials by reducing immune components. Compared to other tissues, the cartilage ECM is dense and difficult to decellularize (Gong et al., 2011). Insufficient decellularization easily leads to excessive residual immunogenic components. While intense decellularization will result in damage to the ECM ultrastructure and the loss of composition (Gilpin and Yang, 2017; Shen et al., 2020). Both run counter to the purpose of decellularization. Although a large number of studies have claimed to be able to achieve effective decellularization, there are still residual cellular constituents (Elder et al., 2010; Luo et al., 2015; Roth et al., 2017; Ghassemi et al., 2019; Bordbar et al., 2020). Effectively balancing the removal of immune components with the destruction of the ECM ultrastructure (or the loss of ECM components), and determining the impact of residual substances on host-biomaterial interactions remain long-debated topics. There is still a large gap in the understanding of the innate and adaptive immune responses resulting from the application of dECM, as well as the role of macrophages and other immune cells in the remodeling of dECM.

Residual cellular material within the ECM may contribute to cytocompatibility problems, and some even led to acute or chronic inflammation (Brown et al., 2009; Nagata et al., 2010; Zhang et al., 2010). The probability of such adverse events can be reduced to some extent through decellularization. Currently, there are no uniform criteria for measuring the degree of decellularization. Most researchers have accepted the following minimum criteria to satisfy the intent of tissue decellularization: (1) the content of double-stranded DNA (dsDNA) should be <50 ng per mg ECM dry weight; (2) the length of DNA fragments should be <200 bp; and (3) staining with 4′,6-diamidino-2-phenylindole (DAPI) or H&E should indicate the lack of visible nuclear material in tissue sections (Crapo et al., 2011). Badylak et al. (2008) proposed these conditions based on the outcomes of research in which an *in vivo* constructive remodeling process was observed, while adverse events were avoided. Of course, these guidelines are not sacrosanct and can differ depending on tissue type, implantation site and host immune function. There is still a lack of a clear scientific basis to establish optimal criteria, and most studies based on decellularization have been evaluated according to the universal criteria described above. The success or failure of any implanted material is related to the host immune system, and host innate or adaptive immune cells will respond to the cellular content, DNA and other immune components of the ECM (Badylak and Gilbert, 2008; Keane and Badylak, 2015).

Residual immunogenic substances in biomaterials resulted from mildly or inadequately decellularized matrix may have proinflammatory effects, which are associated with poor tissue remodeling outcomes (Brown et al., 2009). The focus on nucleic acids in the criteria for decellularization is reasonable, as residual DNA is directly associated with adverse host reactions (Zheng et al., 2005; Nagata et al., 2010). Numerous commercial products composed of dECM are now available for clinical use, such as Restore^®^, GraftJacket^®^, TissueMend^®^, Oasis^®^, and Alloderm^®^. Although most commercial products still contain DNA, the effect of the clinical application of these products is positive to a large extent. The residual DNA content is below 50 ng/mg ECM dry weight, or even lower, which is not enough to cause adverse host reactions that interfere with tissue remodeling (Gilbert et al., 2009).

Residual DNA is usually present in small fragments, which reduces the likelihood that it will play any substantial role in an adverse tissue remodeling response. In the majority of biomaterials used in the clinic, residual DNA consists of fragments that are less than 300 bp in length, and seems to be too short to be of concern. In addition to the low content and short length of residual DNA, degradation of the DNA along with the ECM is another important reason that undesirable host reactions can be avoided *in vivo*, especially for some uncross-linked ECM materials that degrade rapidly. One of the important reasons why DNA fragments are required to be less than 200 bp as a minimal criterion of decellularization may be that the chromosomal DNA of apoptotic cells is autonomously degraded into 180 bp nucleosomal units by caspase-activated DNase (CAD). This DNA is then phagocytosed by macrophages and further degraded by deoxyribonuclease II (DNase II) in lysosomes (Kawane et al., 2003). The accumulation of undigested 180 bp fragmented DNA in macrophages causes them to produce proinflammatory cytokines, such as IFN-β and TNF-α (Yoshida et al., 2005; Kawane et al., 2006). TNF-α may be linked to the development of polyarthritis (Kawane et al., 2006). Additionally, residual DNA is recognized by macrophages as a DAMP through TLRs, especially TLR9 (Hemmi et al., 2000; Zhang et al., 2010; Ohto et al., 2015). Related studies have shown that macrophages can be activated by DNA fragments as small as 24 bp (Karayel et al., 2009; Aamodt and Grainger, 2016). Thus, the current threshold for residual DNA in decellularized tissues does not seem to be ideal, and it serves more as a benchmark established to aid research. As innate immune cells, macrophages participate in the host response to biomaterials in the early stage, and the phenotype of macrophage is significantly associated with tissue remodeling. As mentioned previously, DNA may be one cause of skewed polarization toward an M1 proinflammatory phenotype (Brown et al., 2009). Study found that more M1 proinflammatory macrophages adsorbed onto the dECM scaffold more residual content or longer DNA fragments, while the surroundings of the scaffold from which DNA was effectively eliminated were dominated by M2 anti-inflammatory and remodeling macrophages (Keane et al., 2012). However, what is puzzling is the paucity of research on the interaction between macrophages and residual DNA in dECM. Obviously, there is a large gap in the understanding of the role of these molecules in the host response to dECM.

An in-depth investigation of macrophage-dECM interactions would be helpful in designing tissue engineering scaffolds more scientifically and rationally, thus enhancing the reparative effect and optimizing performance; further research is urgently needed.

## Role of Macrophages in Ecm-Mediated Tissue Regeneration

### Critical Regulator and Effector Cell in Host-Biomaterial Interaction

In tissue engineering, the host response to biomaterials is a key determinant of the success or failure of constructive tissue remodeling. The repair of damaged tissues using biomaterials is a complex process that involves the interaction of diverse immunological and biological systems. These activities do not occur randomly but as a series of finely regulated steps and events, which are correlated with the emergence of different cell types during distinct stages. The repair can be defined as wound healing, which is traditionally divided into many complex and overlapping stages, including coagulation and hemostasis, inflammation, proliferation and remodeling. Similarly, the host response to an implanted biomaterial involves the same steps observed in these stages. The ideal biomaterial should be able to modulate the different stages of the healing response by inducing a transition from inflammation and scar tissue formation to constructive remodeling and functional tissue recovery (Waters et al., 2017). ECM-derived tissue engineering biomaterials, unlike their synthetic counterparts, have been shown to prompt unique and constructive tissue remodeling (Sadtler et al., 2019). ECM has an immunomodulatory effect, particularly in terms of regulating macrophages–the key regulator and effector cells in the host response to biomaterials (Slivka et al., 2014; He et al., 2018). The phenotypical response of macrophages is a key factor in determining the outcome of downstream tissue remodeling (Brown et al., 2012b).

As previously described, macrophages begin to infiltrate in the early stage after implantation and become the primary immune cells in the host response to ECM-derived scaffolds after approximately 3–4 days (Brown et al., 2009). Recently, macrophages have gained extensive attention and been researched, and they are considered to be most relevant to tissue repair mediated by ECM-derived scaffolds. Previous studies have demonstrated the importance of macrophages in tissue reconstruction and regeneration, especially in applications involving tissue engineering biological scaffolds (Linares et al., 2016; Dai et al., 2018b, 2020; Zhang et al., 2020). Specifically, the process of ECM-mediated tissue remodeling depends on the activation of host macrophages toward the M2 phenotype, with anti-inflammatory and immunomodulatory functions (Dearth et al., 2016). At early points, a larger M2/M1 macrophage phenotype ratio predicts the later development of favorable organizational reconstruction (Brown et al., 2012a). Understanding the immunomodulatory effect of biological scaffolds provides potential guidance for the further development of ideal biomaterials to promote tissue repair and regeneration. Unfortunately, to date, the underlying mechanisms by which ECM-derived scaffolds modulate the macrophage phenotype shift from M1 to M2 are largely unknown.

### Phenotypic, Function and Plasticity Features of Macrophages

Macrophages are a heterogeneous cell population with various functional phenotypes that participate in a variety of biological processes, including tissue homeostasis, inflammation, disease progression, and functional reconstruction. Human peripheral blood monocytes can be differentiated into macrophages (M0) and then polarized to different phenotypes. Macrophage phenotypes have been classified along a spectrum ranging from M1 (classically activated, proinflammatory) to M2 (alternatively activated, anti-inflammatory, immunomodulation, remodeling), with multiple subclasses (Martinez and Gordon, 2014; Alvarez et al., 2016). The nomenclature is similar to that used for Th1/Th2 lymphocytes (Mills et al., 2000). These diverse phenotypes can be distinguished by cell surface markers (CD molecules), secreted cytokines and effector molecules, and the metabolism of arginine (Anderson and Jones, 2007). M1 macrophages, the classical proinflammatory phenotype, are known to be activated by IFN-γ alone or in combination with LPS or GM-CSF (Tarique et al., 2015). These cells are able to secrete a series of proinflammatory factors, including TNF-α, IL-1β, IL-6, IL-12, and IL-23 (Lopa et al., 2015). Additionally, M1 macrophages can produce reactive oxygen species (ROS) and present antigens and are inducers and effectors of the Th1 inflammatory response (Mosser, 2003). M1 macrophages produce high levels of inducible nitric oxide synthase (iNOS), leading to the metabolism of arginine into nitric oxide (NO). In contrast, “alternatively activated” M2 macrophages are anti-inflammatory and promote constructive tissue remodeling. M0 macrophages are polarized to the M2 phenotype by exposure to a variety of signals, including the cytokines IL-4 (classical M2-polarizing factor) and IL-13, immune complexes, and matricryptic peptides released during the degradation of ECM-derived scaffolds (Sicari et al., 2014). M2 macrophages are characterized by the secretion of IL-10, chemokine (CCL)-1, CCL-18, and TGF-β (Fahy et al., 2014; Manferdini et al., 2017), the expression of high levels of scavenger, galactose and mannose receptors (e.g., CD206), and promotion of the Th2 response (Mills et al., 2000). Unlike the metabolism of arginine in M1 macrophages, in M2 macrophages, highly expressed arginase (Arg-1) metabolizes it to ornithine and polyamines instead of NO. According to its specific markers and functions, the M2 subphenotype can be further divided into four different subclasses (M2a, M2b, M2c, and M2d) (Ryszer, 2015; Yue et al., 2017), which are often overlooked and regarded as a single group. The functions of the M2 subtypes and others are shown in Table 1. Table 1 summarizes information related to the M1 and M2 phenotypes, including the inducers, expressed markers, secreted molecules and functions.

**TABLE 1:** Summary of macrophage subtypes and functions.

**Macrophage phenotype**		**Inducers**	**Expressed markers**	**Secreted molecules**	**Functions**	**References**
M1		IFN-γ, LPS, GM-CSF	CD80, CD86, CD68, CCR7	TNF-α, IL-1β, IL-6, IL-12, IL-23, iNOS, ROS, MMPs, VEGF	Proinflammatory, tissue damage, Th1-type reaction	Mosser, 2003; Verreck et al., 2004; Lopa et al., 2015; Tarique et al., 2015; Qiu et al., 2018
	M2a	IL-4, IL-13	CD206, CD163	FIZZ1, Arg-1, TGF-β, CCL-18	Tissue repair and remodeling, anti-inflammatory	Spiller et al., 2014; Raimondo and Mooney, 2018; Wiktorowicz et al., 2019
	M2b	ICs, LPS, IL-1β, TLR ligand	IL-10R, CD86, CD163	IL-10, IL-1β, IL-6, TNF-α	Immunoregulation, homeostasis	Gerber and Mosser, 2001; Yue et al., 2017; Yang et al., 2019
M2						
	M2c	IL-10, TGF-β, glucocorticoid	CD163, CD206	TGF-β, Arg-1, CCL-16, CCL-18, MMPs	Pro-wound healing, matrix deposition, tissue remodeling	Zizzo et al., 2012; Spiller et al., 2014; Waters et al., 2017; Becker et al., 2018
	M2d	TLR ligand, adenosine	VEGF, IL-12^*low*^	IL-10, VEGF	Angiogenesis	Grinberg et al., 2009; Ferrante and Leibovich, 2012

*IL, interleukin; IFN-γ, interferon-gamma; CD: cluster of differentiation; ICs: immune complexes; LPS, lipopolysaccharide; TLR, Toll-like receptor; TGF-β, transforming growth factor-beta; iNOS, inducible nitric oxide synthase; TNF-α, tumor necrosis factor-alpha; CCL, chemokine ligand; ROS, reactive oxygen species; GM-CSF: granulocyte-macrophage colony stimulating factor, Th1: type 1 helper; MMPs, matrix metalloproteinases; CCR, chemokine receptor; FIZZ1: Found in inflammatory zone 1; Arg-1, arginase-1; VEGF, vascular endothelial growth factor.*

### Promote Tissue Regeneration by Modulate Macrophage Polarization

Significant effort has been made to regulate macrophage polarization using biomaterial and scaffold design strategies to promote tissue regeneration, which is known as immuno-informed techniques. It is hoped that the development of biomaterials that can stimulate the polarization of macrophages toward reparative functional phenotypes is becoming a research hotspot (Spiller et al., 2015). Designing a biomaterial with immunomodulatory function is a practical and efficient strategy. One study investigated the immunomodulatory properties of fabricated biomimetic with a bone-like staggered nanointerface during bone regeneration, and it was found that the hierarchical nanointerface possess the ability to facilitated M2 macrophage polarization to promote endogenous bone regeneration (Jin et al., 2019). Controlling the macrophage polarization by manipulating the nanomorphology of biomaterial surface may be an effective way to improve the performance of biomaterials (Ma et al., 2014). MSCs exosomes-mediated response in cartilage repair was also found to be associated with regenerative M2 macrophages (Zhang C.H. et al., 2018). In designing an immune-informed regenerative biomaterial, the most targeted approach to modulate macrophage polarization is to release factors (such as IL-4, IL-10) that direct polarization, and this can be achieved through a controlled release system (Sridharan et al., 2015; Li M. et al., 2020).

Extracellular matrix-based scaffolds exhibit such immunoregulation potential, and the resulting constructive remodeling response is associated with a timely transition of the macrophage phenotype from proinflammatory M1 to immunomodulatory and constructive M2 (Sicari et al., 2014; Wu et al., 2019). Both the classically and alternatively activated macrophage phenotypes are transient and difficult to define, which means that macrophages can polarize into diverse phenotypes based on changes in the microenvironment (Tarique et al., 2015). The microenvironment in which biomaterials are implanted *in vivo* is complex, and macrophages are exposed to a variety of stimuli, including various cytokines and effectors secreted by host immune cells (Kimura et al., 2016), immune components, biochemical cues (e.g., surface chemical and composition) (Palmer et al., 2014; Hotchkiss et al., 2018) and biophysical cues (e.g., topography, stiffness, and pore size) (Madden et al., 2010; Bartneck et al., 2012; Abaricia et al., 2020) of biomaterials or scaffolds, and degradation products (Sicari et al., 2014). The activation and phenotypical polarization of macrophages *in vivo* is modulated by multiple factors. These factors should be taken into account in the design of future tissue engineering bioscaffolds to regulate the host response in a direction that favors constructive tissue remodeling (e.g., polarization of macrophages toward an M2 phenotype) (Garg et al., 2013; Alvarez et al., 2016).

The implantation of a biomaterial can induce a host response, which determines the outcome of constructive remodeling of the damaged tissue. The mechanism of ECM derived scaffolds mediate immunomodulation (e.g., the macrophage phenotype shifts from M1 to M2) *in vivo* is still unclear (Mimura et al., 2016; Huleihel et al., 2017). Moreover, not all ECM scaffolds derived from diverse source tissues can induce this phenomenon. The effect of ECM scaffolds derived from eight different source tissues (all porcine) on the polarization of macrophages was investigated. Macrophages exposed to ECM from the small intestinal submucosa, urinary bladder, brain, esophagus, and colon expressed a predominantly M2 phenotype. Conversely, macrophage exposure to dermal ECM resulted in a predominantly M1 phenotype, whereas liver ECM and skeletal muscle ECM did not significantly change the phenotype of the macrophages (Dziki et al., 2017). It is notable that some studies on regulation of the macrophage phenotype by ECM have conflicting results (Meng et al., 2015). Different decellularization protocols are applied to each tissue, which makes the analysis more complicated. In addition, unique ECM components from diverse source tissues make the seemingly contradictory results somewhat reasonable. Despite the marked differences and heterogeneity among various macrophage subtypes, their phenotype is highly plastic and dynamic (Hume, 2015). In fact, macrophage phenotypes exist in a range, with M1 and M2 as extreme values. Any given cell can express or co-express characteristics of the M1 or M2 phenotype, rather than exhibit a discrete functional state with a well-defined boundary (Mosser and Edwards, 2008). The description of macrophage activation and polarization is currently controversial and confusing, and the lack of a consensus diminishes the reference value of research and hinders progress to some extent. A guideline, published in 2014, will contribute to the better unification of experimental standards in future research (Murray et al., 2014). Although there is evidence that it is too simple to classify activated macrophages as M1 or M2, many studies in the literature have adopted these categories to illustrate specific findings. The simple classification of macrophages into M1 and M2 is inadequate, but it is useful for examining the inflammatory phenotype in response to biomaterials, especially when measuring the levels of inflammatory cytokines. The process of ECM-derived scaffolds facilitating tissue repair by regulating macrophage phenotypical transformation is complex. A clear and detailed report on distinguishing macrophage phenotypes will help analyze and understand this process. Modulating the components of the immune system to promote tissue regeneration. Once we have a clear understanding of host-biomaterial interactions, it will be possible to construct suitable immuno-informed decellularized tissue that can control tissue remodeling and initiate beneficial reprogramming *in vivo* (Taraballi et al., 2018).

## Articular Cartilage ECM (AC-ECM)

### Rich in Collagen

Articular cartilage ECM (AC-ECM) is a biomaterial widely studied in cartilage tissue engineering at present. It has good biocompatibility and is very similar to the natural cartilage matrix in terms of composition. AC-ECM scaffolds support cell adhesion and proliferation and are able to recruit autologous endogenous stem cells and induce their differentiation toward chondrocytes *in vivo*, allowing them to maintain the chondrocyte phenotype and thus achieving the *in situ* regeneration of cartilage (Xue et al., 2012; Almeida et al., 2016; Nasiri and Mashayekhan, 2017; Li et al., 2019a). The main components of AC-ECM are Col II and proteoglycans, and Col II accounts for over 80% of the dry weight of AC-ECM. The collagen network is essential in determining the mechanical properties of cartilage, and small amounts of other collagen types (I, III, V, VI, IX, X, and XI) also exist in AC-ECM (Gannon et al., 2015a, b; Campos et al., 2019; Wang et al., 2020a). Col II plays a vital role in the development and maturation of chondrocytes. It has an immunomodulatory effect, relieving the degeneration of cartilage matrix in OA by inhibiting the STAT1 signaling pathway of proinflammatory macrophages (Dai et al., 2018a). Macrophages express prochondrogenic cytokines, which stimulate chondrocytes to secrete matrix components while activating glycine receptors and reducing the intracellular calcium concentration to inhibit chondrocyte apoptosis and hypertrophy (Dai et al., 2018b). In short, both AC-ECM and Col II-based engineering scaffolds have great potential for the repair of damaged cartilage.

However, what is often overlooked and worrying is that Col II has immunogenicity and can induce arthritis by emulsification with an adjuvant, which is known as collagen-induced arthritis (CIA) (Trentham et al., 1977). The following discussion of this problem is mainly focused on the frequently studied AC-ECM scaffold.

### Antigenic and Immunogenic Responses to Collagen

Before discussing the immunochemical property of any protein, it is important to draw a distinction between the potentially ambiguous terms “antigenicity” and “immunogenicity.” Although it is difficult to make such a distinction due to the presence of a number of extrinsic factors, and for the purpose of the present discussion, the following simple distinction is adopted: antigenicity will be used to refer to the ability of a substance to interact with antibodies or cellular receptors, whereas immunogenicity will be used to refer to the ability to induce an immune response–a process that includes antibody synthesis and interaction. Therefore, we can think of any biomaterial that has immunogenicity to also have antigenicity.

Collagen is considered to be a safe and multifunctional biomaterial, but it has also raised concerns regarding its potential to elicit an immune response. Although the clinical incidence of adverse reactions to collagen implants is low, this do occur. Previously, it was widely accepted that collagen is an inert protein and does not have immunogenicity. Later studies have shown that it can interact with antibodies, but still regarded as a weak antigen. In tissue engineering, the interpretation of the immunogenicity of collagen-based scaffolds is often complicated by the presence of other non-collagen components, including cellular and nuclear contents, MHCs, and alpha-Gal epitopes, among others (Wong and Griffiths, 2014; Sutherland et al., 2015). Currently, few studies have targeted the immunological properties of collagen. Due to most collagen-based scaffolds were composed of type I collagen (and a small amount of type III collagen), which is generally not thought to evoke a potentially adverse host immune response (Bayrak et al., 2013; Shetty et al., 2013; Yuan et al., 2014). However, as research progresses to address the issue of cartilage repair and regeneration, AC-ECM (enriched with Col II) and marine collagen (extracted from marine organisms) have shown good prospects for application and thus received increasing attention from researchers (Pustlauk et al., 2016; Tamaddon et al., 2017; Dai et al., 2018b). Allogeneic or xenogeneic Col II can induce arthritis (i.e., CIA) when emulsified with an adjuvant. Therefore, the immunological property of collagen has once again raised concern, which will be described in detail below.

Trentham et al. (1977) first discovered that emulsified Col II could induce arthritis and established an experimental arthritis model–a CIA model (Trentham et al., 1977). CIA is the most extensively studied model of rheumatoid arthritis (RA), mainly involving distal joints (especially the posterior ankle joint) (Wu et al., 2015). Its pathological features are similar to those of RA, including synovial hyperplasia, inflammatory cell infiltration, and cartilage destruction (Scarneo et al., 2019; De et al., 2020). Col II is the major protein in the cartilage of joints targeted by RA, and anti-Col II autoantibodies are present in the serum of RA patients, which indicates that Col II can induce an autoimmune response of an arthritic nature in the body (Manivel et al., 2015). There is some evidence that antibodies in RA patients target the same Col II molecular regions as in those in CIA (Burkhardt et al., 2002). In addition, the presence of T and B cell immunity has also been reported (Dimitrijević et al., 2020), but it is not clear whether this is a pathogenic factor or result of RA. Recent studies have also shown that the immune response may be involved in the development of OA. When inflammation occurs on the surface of articular cartilage for some reason, various components (mainly Col II) are exposed and thus stimulate B lymphocytes to produce anti-Col II antibodies, resulting in an immune response in OA. Therefore, as a biomaterial in cartilage tissue engineering, whether AC-ECM rich in Col II will cause an intraarticular immune reaction has become a concern. The mechanism of CIA is still unclear, but it is certainly inextricably linked to the structural characteristics of Col II.

**FIGURE 2:**
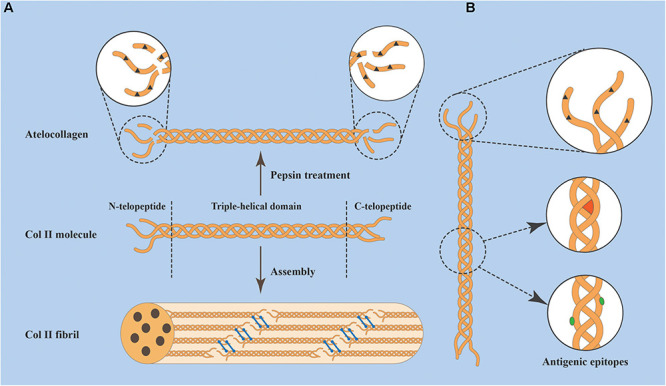
**(A)** Three α_1_ chains intertwine to form a type II collagen (Col II) molecule, which consists of three parts: a triple-helical domain and N- and C-telopeptides. Collagen molecules spontaneously assemble into fibrils head to tail in a quarter-stagger array and are stabilized by covalent bonds (blue bars). The molecule can be treated with pepsin to remove (not completely remove) telopeptides, yielding Atelocollagen. **(B)** Antigenic epitopes of Col II include those located within the telopeptides (dark brown triangles), those located in the helical region and dependent on the conformation (red sector), and those located in the helical region and dependent on the amino acid sequence (green ovals).

A Col II molecule consists of three identical α_1_ chains (a homotrimer), each containing 1487 amino acids with a molecular weight of approximately 130 kDa (Miller, 1971). Each of the three α_1_ chains forms a left-handed helix by itself and then further intertwines to form a right-handed triple-superhelical structure, which is the main and unique structural region of the molecule, known as the “triple-helical domain.” The greatest feature of the “triple-helical domain” is the periodically repeated arrangement of amino acids presenting [Gly-X-Y]_*n*_, in which the positions of X and Y are usually occupied by proline (Pro) and hydroxyproline (Hyp), respectively (Canty and Kadler, 2005). Each chain has a short peptide extension at each end of the helix, the telopeptide (i.e., the amino (N)- and carboxyl (C)-telopeptides), which is a non-helical structure and does not contain Gly-X-Y repeats (Liu et al., 2015). The telopeptide region determines the intermolecular interactions that contribute to (and stabilize) normal fiber assembly. The amino acid sequences of telopeptides may vary from species to species, while certain cross-linked regions involved in fiber formation are highly conserved. Once secreted into the ECM, collagen molecules will be arranged head to tail in a quarter-stagger array and then covalently cross-linked into fibers through polymerization and disulfide bonds, forming the skeleton of the cartilage matrix. The structures of the Col II molecule and assembled fibrils are shown in Figure 2A.

The antigenic epitopes that determine the immunogenicity of Col II can be divided into three main categories: (1) those located the non-helical terminal regions (telopeptides), existing in both natural and denatured collagen; (2) those dependent on the amino acid sequence of the helical region of the molecule, exposed after this region is unwound and thus present in denatured collagen; and (3) those dependent on the triple-helical conformation, which only exist in natural collagen as the integrity of the triple helix must be maintained (Figure 2B). Sequence homology is highly conserved in the helical portion of Col II among different species, and the degree of change in the amino acid sequence is less than a few percent. Hidden epitopes (such as amino acid sequences in the helical region) interact with antibodies when the triple helix is unwound (Dodge and Poole, 1989). This fact may have implications for the host immune response to AC-ECM implants as they denature and degrade. The degree of telopeptide variation is much greater, which is considered to be the main determinant of Col II immunogenicity. Some studies have tried to reduce the immunogenicity by using proteolytic enzymes (e.g., pepsin) to remove the terminal telopeptides, resulting in what is known as Atelocollagen (Lin and Liu, 2006; Jeevithan et al., 2015; Figure 2B). However, there is still a lack of scientific evidence regarding the effect of this treatment on collagen immunogenicity. It should be made clear that pepsin treatment does not completely remove telopeptides. Furthermore, although this process cannot disrupt the triple-helical structure, the destruction of fiber networks will reduce the mechanical properties of collagenous implants (Shayegan et al., 2016). In the absence of a clear immunological benefit obtained by Atelocollagen, the other two types of antigenic epitopes of collagen molecules cannot be ignored.

It should be clarified that Col II-induced arthritis (CIA) develops slowly with the aid of an adjuvant, thus stimulating a high and persistent antibody response, which can be interpreted as an amplification effect. AC-ECM derived scaffolds usually undergo cross-linking, which delays the degradation of the scaffold and appears to have an effect similar to that of adjuvant. Col II is capable of inducing CIA in different species, but the sensitivity to Col II varies among species. Despite the fact that some implants containing Col II did not elicit adverse immune reactions in animal models (Nehrer et al., 1998; Mainil-Varlet et al., 2001), none of these studies were conducted in species previously shown to be susceptible to CIA. Although there is no evidence to support the theory that the implantation of biomaterials containing Col II could induce autoimmunity, there is also no direct evidence to disprove this theory.

### Discussion of Potential Immune Response to AC-ECM

Articular cartilage ECM derived scaffolds are typically obtained in two ways: the granulation of cartilage by chopping or crushing, followed by decellularization and cross-linking to produce a highly porous customized scaffold structure, but at the expense of mechanical properties (Yang et al., 2008; Browe et al., 2019). Or the decellularization of intact cartilage explants without complete disruption of the collagen network, with relatively better mechanical properties but a very low porosity that limits the infiltration of cells (Schwarz et al., 2012; Li et al., 2019c). To some extent, these two kinds of scaffolds may alter the immune response induced by Col II. The purification of commercial collagen products is usually conducted at 4°C to prevent denaturation, a requirement apparently not met in the preparation of AC-ECM derived scaffolds. Whether Col II in AC-ECM derived scaffolds retains its natural structure, whether the decellularization process leads to the exposure of antigenic epitopes and whether cross-linking can hide antigenic epitopes remains unknown. In contrast to pure Col II-derived scaffolds, AC-ECM derived scaffolds contain other components such as GAGs that wrap around Col II, which may hide its antigenic determinants. These questions remain to be answered by follow-up research. An in-depth understanding of the immunological properties of Col II-containing scaffolds and their impact on cartilage will lay a foundation for the more scientific design of optimal scaffolds for use in cartilage tissue engineering.

## Concluding Remarks and Future Perspectives

Nowadays, a wide variety of biomaterials are used for the repair of cartilage and other tissues in tissue engineering. However, most of biomaterials do not function as expected, or some even lead to the occurrence of adverse events. These unsatisfactory results are mainly due to the adverse interaction between the implanted biomaterials and the host’s immune system. The host-biomaterial interaction is crucial to determining the outcome of downstream tissue reconstruction. It is influenced by numerous factors, such as immune components of material, cytokines and inflammatory agents induce by implants. Macrophages are the critical immune regulators, and can promote inflammatory or facilitate repair and regeneration attributing to their plasticity and versatility. The relationship between tissue healing and the immune system is very complex, since immune components of material or the cells involved could drastically change the repair outcome. Modulating the immune system through immunomodulatory biomaterials to promote repair is an effective strategy in future tissue engineering.

In recent years, Col II-containing scaffolds, especially AC-ECM-derived scaffolds, have become a hot research topic. It is worth noting that Col II has immunogenicity and could induce arthritis (i.e., CIA), an issue that is easily overlooked by the public and has attracted our attention. In this paper, the immunological characteristics of Col II are analyzed, and the potential immunogenicity of Col II-containing biomaterials is summarized.

An in-depth exploration of the host immune response to biomaterials not only is critical to understanding the repair mechanisms of these biomaterials but also paves the way for the development of ideal cartilage repair biomaterials in the future. All kinds of biomaterials have been explored for their potential in cartilage repair, and some have shown good results, which largely depend on the host response to the implanted biomaterial. Biomaterials have been designed with a keen interest in the development of “passive biomaterials” to limit undesirable immune responses. Emphasis has been placed on promoting repair by preventing (or reducing) protein adhesion to the surface of the implanted biomaterial and the resulting inflammatory/immune cell activation and interaction. However, researchers have come to realize that the immune system plays a fundamental role in coordinating and defining the nature of the repair process. Allowing specific biological reactions is helpful for biomaterial-mediated remodeling. The link between the host response and tissue repair is complicated, and studies of host-biomaterial interactions have mainly focused on macrophages. The concept of the optimal biomaterial is shifting from reducing the host response to triggering the desired immune response, thereby facilitating constructive remodeling. With the continuous development of tissue engineering and regeneration medicine, regulating the interaction between biomaterials and the host must be a key part of the design and a focus of future work in this field. This future work will lead to developments that will hopefully promote the clinical translation of biomaterials.

## Author Contributions

All authors listed have made a substantial, direct and intellectual contribution to the work, and approved it for publication.

## Conflict of Interest

The authors declare that the research was conducted in the absence of any commercial or financial relationships that could be construed as a potential conflict of interest.
